# Supplementary material for: Hardening and Strain Localisation in Helium-Ion-Implanted Tungsten
Source: Sci Rep. 2019 Dec 4;9:18354. doi: 10.1038/s41598-019-54753-3 (PMC6892934; doi:10.1038/s41598-019-54753-3)
Supplement: Supplementary file 1 — Supplementary Material [file 41598_2019_54753_MOESM1_ESM.pdf]

Supplementary Material: Hardening and Strain Localisation in Helium-Ion-Implanted Tungsten

Suchandrima Das[a,1], Hongbing Yu[a], Edmund Tarleton[a,b,2], Felix Hofmann[a,3]

[a]*Department of Engineering Science, University of Oxford, Parks Road, Oxford OX1 3PJ, UK*
[b]*Department of Materials, University of Oxford, Parks Road, Oxford OX1 3PH, UK*

[1]suchandrima.das@eng.ox.ac.uk

[2]edmund.tarleton@eng.ox.ac.uk

[3]felix.hofmann@eng.ox.ac.uk

Appendix A - Determination of the value of $\boldsymbol{\sigma}_{xx}^{BC}$ and $\boldsymbol{\sigma}_{yy}^{BC}$

The ε_{zz}^{dev} component of the deviatoric lattice strain in the helium-implanted sample, as measured by the white-beam Laue diffraction, is ~ 550 × 10^{-6}. This value was obtained by averaging the measured ε_{zz}^{dev} over 1.5 µm of the helium-implanted layer. The ε_{zz} component of the total strain tensor was then computed from ε_{zz}^{dev}. The total strain tensor, $\boldsymbol{\varepsilon}^{e}$, is related to the deviatoric component $\boldsymbol{\varepsilon}_{dev}^{e}$ as

$$\boldsymbol{\varepsilon}^{e} = \boldsymbol{\varepsilon}_{dev}^{e} + 1/3\ \mathrm{Tr}\,(\boldsymbol{\varepsilon}^{e})\boldsymbol{I} \qquad \text{(A.1)}$$

where $\boldsymbol{I}$ is the identity matrix. The ε_{xx} and ε_{yy} components of the total lattice strain tensor are expected to be zero, as deformation along X and Y directions is restricted in order to maintain geometrical continuity between the implanted layer and the substrate [1]. Thus $\mathrm{Tr}\,(\boldsymbol{\varepsilon}^{e}) = \varepsilon_{zz}$. Therefore, Eq. (A.1) can be re-written as

$$\varepsilon_{zz} = \varepsilon_{zz}^{dev} + 1/3\ \varepsilon_{zz} \qquad \text{(A.2)}$$

$$\varepsilon_{zz}^{dev} = 2/3\ \varepsilon_{zz} \qquad \text{(A.3)}$$

Using Eq. (A.3), $\varepsilon_{zz} = 825 \times 10^{-6}$.

ε_{zz} can be related to the "correctional boundary conditions" $\sigma_{corr_{xx}}$ and $\sigma_{corr_{yy}}$, generated to

counteract the helium-implantation induced eigenstrain, in order to maintain geometrical continuity between the implanted layer and the substrate [1].

$$\sigma_{corr_{xx}} = \frac{-E\varepsilon_{zz}}{3(1+\nu)} = \sigma_{corr_{yy}} \qquad \text{(A.4)}$$

Detailed derivation of Eq. (A.4) can be found elsewhere [1]. Knowing, ε_{zz}, the stress boundary conditions, $\sigma_{corr_{xx}} = \sigma_{corr_{yy}}$ or what is here referred to as $\sigma_{xx}^{BC} = \sigma_{yy}^{BC}$ are computed to be -260 MPa.

Appendix B: Comparison of CPFE with Continuum-mode FEM

The CPFE predicted surface pile-up around the nano-indent in the helium-implanted material agrees very well with the AFM measurements quantitatively and in terms of pile-up patters (Figure 2 in main text). CPFE uniquely allows this prediction of the deformation pattern by accounting for crystallographic slip. A continuum plasticity simulation, performed on the same model, is unable to reproduce the orientation dependence of pile-up as shown in Figure B.1.

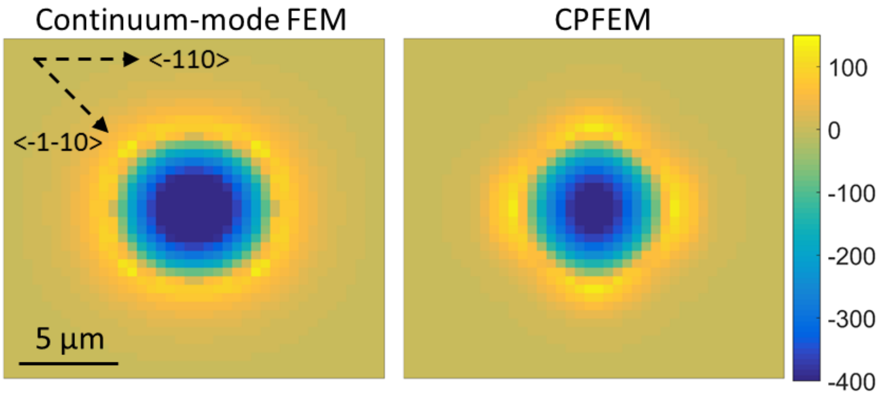

Figure B.1 – Surface pile-up around nano-indents in 001-oriented helium-implanted tungsten single crystal as predicted by continuum-based simulation (left, which does not account for crystal orientation) and by CPFE (right). The colour bar shows surface height in nm.

Appendix C

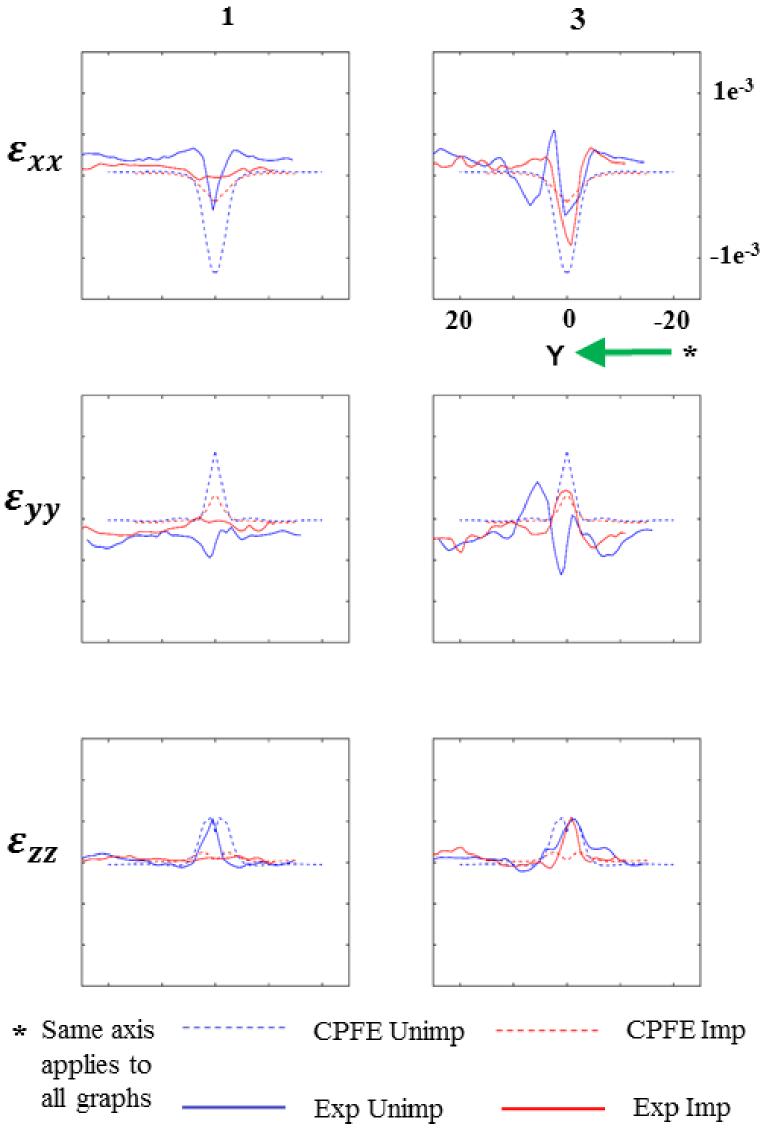

Figure C.1 - Line plots corresponding to the contour plots in Figure 5 (c) and (d) in the main text. Direct lattice strains are plotted along a horizontal line 4.5 µm below the indent (shown by dotted white lines in Figure 5 (a) and (b) in main text). Slices 1 and 3 correspond to YZ sections drawn 5 µm away from the indent centre, on either side, along the X-axis.

Appendix D

List of 13 ion energies used and the corresponding fluence for the helium ion implantations:

Ion Energy (MeV)	3000 appm He
	Fluence (ions/cm^2)
0.05	2.40E+15
0.1	1.80E+15
0.2	4.20E+15
0.3	1.20E+15
0.4	4.80E+15
0.6	5.20E+15
0.8	5.00E+15
1	5.00E+15
1.2	5.00E+15
1.4	5.00E+15
1.6	5.50E+15
1.8	7.00E+15
2.0	5.00E+15

Table D.1 - List of 13 ion energies used and the corresponding fluences for the helium ion implantations.

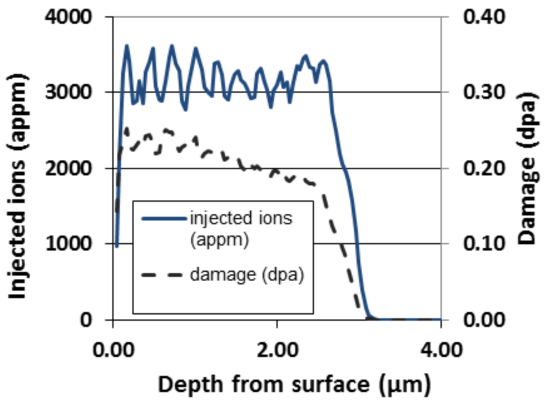

Figure D.1 - Helium-implantation profile as estimated by the SRIM code [2].

Appendix E – Details of the CPFE Model

The sample block was assigned elastic properties of tungsten and the indenter was approximated as a discrete rigid wire frame (to avoid a full meshing and increase in simulation size). Contact between the sample and indenter was defined using the Abaqus node to surface contact algorithm and the contact was considered to be frictionless as past studies have shown that the mechanical response of the underlying substrate is not significantly affected by the coefficient of friction [3]. The results of indentation load reached were scaled with an effective modulus E_{eff} to account for the indenter tip compliance [4].

$$P\ (exp.) = \frac{E_{eff\ (exp.)}}{E\ (FEA)}\ P(FEA) \qquad \text{(E.1)}$$

$E_{diamond}$	$E_{tungsten}$	$\nu_{diamond}$	$\nu_{tungsten}$	E_{eff}	$R_{indenter}$
1143 GPa	410 GPa	0.0691	0.28	322.58 GPa	4.2 µm

Table E.1 – Values of Young's modulus and Poisson's ratio for diamond (indenter tip) and tungsten (indented sample) as obtained from literature [5–7]. With the assumption of isotropic, linear elastic solid, the Young's modulus and Poisson's ratio are related to the elastic constant as follows: $E = c_{11} - 2\left(\frac{c_{12}^2}{c_{11} + c_{12}}\right)$ and $\nu = c_{12}/(c_{11} + c_{12})$.

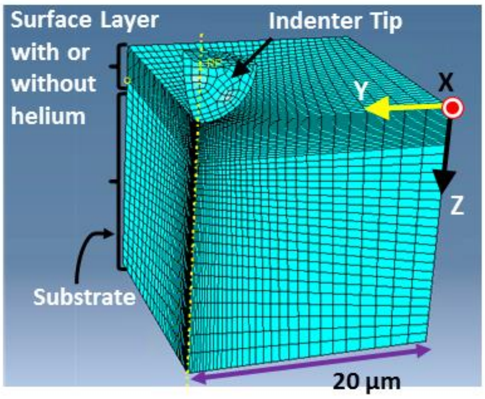

Figure E.1 - Refined mesh for the 3D crystal plasticity finite element simulation of the tungsten sample indented by a 4.2 µm radius spherical indenter with the X, Y, Z coordinate frame superimposed.

Appendix F – UMAT Parameters

**Material Property**	**Value**	**Reference**
Elastic modulus E	410 GPa	[5–8]
Shear modulus G	164.4 GPa	[5–8]
Poisson's ratio υ	0.28	[5–8]
Burgers' vector b	2.7×10^{-10} m	[9]
Stress boundary conditions $\sigma_{xx}^{BC} = \sigma_{yy}^{BC}$	-260 MPa	Appendix A
τ_H^0	750 MPa	Section 4.6.3 in main text & Appendix G
Activation energy ΔF	0.22 eV	Chosen to reduce strain-rate sensitivity
Boltzmann constant k	1.381×10^{-23} J/K	[10]
Temperature T	298 K	Room temperature assumed similar to experimental conditions
Attempt frequency v	1×10^{11} s^{-1}	[11]
Density of statistically stored dislocations, ρ_{SSD}	1×10^{10} m^{-2}	Appendix H
Density of mobile dislocations ρ_m	3.5×10^{10} m^{-2}	Appendix H
Probability of pinning Ψ	0.657×10^{-2}	Value chosen and kept fixed
τ_c^0	360 MPa	Fitted to experimental data of unimplanted sample
γ	0.025	Fitted to experimental data of helium-implanted sample
C'	0.0065	Fitted to experimental data of unimplanted sample

Table F.1– List of parameters used in the constitutive law in the CPFE formulation and their corresponding values.

Appendix G: Determination of the value of τ_H^0

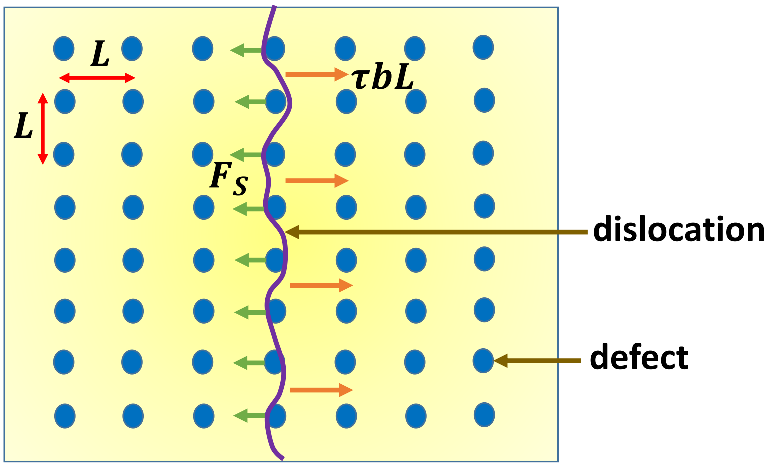

Figure G.1 – View of the slip plane interspersed with helium-implantation induced defects with a dislocation line trying to pass through.

Appendix H: Estimating values for SSD

Laue diffraction measurements performed on nano-indented pure tungsten showed the presence of GND density on the order of ~ 10^{18} m^{-2}. This value agreed well with CPFE predictions of the indentation experiment [17]. The SSD densities assumed here, ρ_{SSD} and ρ_m, for the CPFE calculations were taken to be much smaller than the GND density estimate, on the order of 10^{10} m^{-2}.

References

1. Das, S., Liu, W., Xu, R. & Hofmann, F. Helium-implantation-induced lattice strains and defects in tungsten probed by X-ray micro-diffraction. *Mater. Des.* **160,** 1226–1237 (2018).

2. Das, S. *et al.* The effect of helium implantation on the deformation behaviour of tungsten: X-ray micro-diffraction and nanoindentation. *Scr. Mater.* **146,** 335–339 (2018).

3. Wang, Y., Raabe, D., Klüber, C. & Roters, F. Orientation dependence of nanoindentation pile-up patterns and of nanoindentation microtextures in copper single crystals. *Acta Mater.* **52,** 2229–2238 (2004).

4. Li, M., Morris, D. J., Jennerjohn, S. L., Bahr, D. F. & Levine, L. Finite element analysis and experimental investigation of the Hertzian assumption on the characterization of initial plastic yield. *J. Mater. Res.* **24,** 1059–1068 (2009).

5. Featherston, F. H. & Neighbours, J. R. Elastic constants of tantalum, tungsten, and molybdenum. *Phys. Rev.* **130,** 1324–1333 (1963).

6. Bolef, D. I. & De Klerk, J. Elastic Constants of Single-Crystal Mo and W between 77° and 500°K. *J. Appl. Phys.* **33,** 2311–2314 (1962).

7. Klein, C. A. & Cardinale, G. F. Young's modulus and Poisson's ratio of CVD diamond. *Diam. Relat. Mater.* **2,** 918–923 (1993).

8. Ayres, R. A. *et al.* Elastic constants of tungsten–rhenium alloys from 77 to 298 °K. *J. Appl. Phys.* **46,** 1526–1530 (1975).

9. Dutta, B. N. & Dayal, B. Lattice Constants and Thermal Expansion of Palladium and Tungsten up to 878 C by X-Ray Method. *Phys. status solidi* **3,** 2253–2259 (1963).

10. Sweeney, C. A. *et al.* The role of elastic anisotropy, length scale and crystallographic slip in fatigue crack nucleation. *J. Mech. Phys. Solids* **61,** 1224–1240 (2013).

11. Cottrell, A. *An introduction to metallurgy*. (Universities Press, 1990).

12. Stukowski, A., Cereceda, D., Swinburne, T. D. & Marian, J. Thermally-activated non-Schmid glide of screw dislocations in W using atomistically-informed kinetic Monte Carlo simulations. *Int. J. Plast.* **65,** 108–130 (2015).

13. Weinberger, C. R., Boyce, B. L. & Battaile, C. C. Slip planes in bcc transition metals. *Int. Mater. Rev.* **58,** 296–314 (2013).

14. Po, G. *et al.* A phenomenological dislocation mobility law for bcc metals. *Acta Mater.* **119,** 123–135 (2016).

15. Samolyuk, G. D., Osetsky, Y. N. & Stoller, R. E. The influence of transition metal solutes on the dislocation core structure and values of the Peierls stress and barrier in tungsten. *J. Phys. Condens. Matter* **25,** (2013).

16. NIST Chemistry Web book, SRD69. *National Institute of Standards and Technology* Available at: https://webbook.nist.gov/cgi/cbook.cgi?ID=C7440337&Mask=2.

17. Das, S., Hofmann, F. & Tarleton, E. Consistent determination of geometrically necessary dislocation density from simulations and experiments. *Int. J. Plast.* (2018). doi:10.1016/j.ijplas.2018.05.001
